# Study design of an interdisciplinary and participatory nature-based palliative rehabilitation intervention in a Danish nursing home for people with severe dementia

**DOI:** 10.1186/s12877-022-03513-6

**Published:** 2022-10-23

**Authors:** Tanja Schmidt, Marie Christoffersen Gramkow, Dorthe Varning Poulsen, Louise Holm Miller, Lene Wermuth, Ulrika K. Stigsdotter

**Affiliations:** 1 Research Unit for Active Living, Department of Sports Science and Clinical Biomechanics, University of Southern Denmark, Campusvej 55, 5230 Odense M, Denmark; 2 Section for Landscape Architecture and Planning, Department of Geosciences and Natural Resource Management, University of Copenhagen, Rolighedsvej 23, 1958 Frederiksberg C, Denmark; 3 Department of Neurology, Slagelse Hospital, Ingemannsvej 18, 4200 Slagelse, Denmark

**Keywords:** Palliative rehabilitation, Severe dementia, Garden design, Co-design, BPSD

## Abstract

**Background:**

A limited amount of research has examined how nature-based palliative rehabilitation can be implemented in nursing homes for people with dementia, even though evidence suggests that these gardens are underused. This paper will present the study protocol of an intervention study co-designed in an interdisciplinary collaboration with a nursing home for people with dementia, to develop a tailored nature-based palliative rehabilitation program to increase qualified use of garden with the purpose of promoting a range of health outcomes.

**Methods:**

The study is a single-cased quasi-experimental mixed methods study. The intervention will be developed, designed, and implemented in collaboration with the nursing home, using different co-design tools and methods. The effect of the intervention will be evaluated using the The Neuropsychiatric Inventory Nursing Home version in combination with medication use, a survey on staff burnout, and cameras in the garden to register garden use. A process evaluation with single- and focus group interviews consisting of various stakeholders in the study will be used to gain knowledge on the intervention processes and implementation.

**Discussion:**

The paper presents new approaches in the field of palliative rehabilitation for people with dementia using nursing home gardens, through interdisciplinary collaboration, participatory co-design approach and mixed methods design. Using both effect and process evaluation, the study will provide unique insights in the role and importance of participatory process, interdisciplinary collaboration, and tailoring palliative rehabilitation activities in gardens at nursing homes to local needs and wishes. These results can be used to guide other nursing homes and renewal projects in the future.

**Trial registration:**

ISRCTN, ISRCTN14095773. Registered 15 July 2022—Retrospectively registered.

**Supplementary Information:**

The online version contains supplementary material available at 10.1186/s12877-022-03513-6.

## Background

The continuing increase in life expectancy worldwide has caused an increase in the number of people with dementia (PwD), of which the neurodegenerative disease Alzheimer's is the most common cause. In Denmark, it is estimated that 1.5% of Danish citizens over the age of 65 have dementia, and that this number will increase to 2.6% in 2050 as we live longer [1]. Also, dementia is the fourth leading cause of death in Denmark [2]. The disease is usually associated with a range of behavioral and psychological symptoms called Behavioral and Psychological Symptoms of Dementia (BPSD) leading to functional and cognitive impairment [3]. BPSD include aberrant motor behavior, anxiety, agitation, elation, depression, irritability, apathy, hallucinations, delusions, disinhibition, and changes in sleep or appetite, and is estimated to affect up to 90% of all PwD [4]. A range of BPSD symptoms will commonly appear during the progression of the disease, making it more difficult for PwD to take care of themselves, leading to distress among PwD and their caregivers, institutionalization, long-term hospitalization, and misuse of medication, resulting in increased health care costs [3]. Consequently, the increase in PwD and the burden of BPSD creates great strain on nursing homes and long-term facilities, leading to major challenges for the society, care staff, relatives and the person suffering from the disease [5, 6]. The institutionalization and the experience of not being able to take care of oneself due to reduced functional capacity, often results in reduced quality of life and increased depression in PwD [7, 8], which increases the complexity of care. Caring for PwD is demanding due to BPSD, requiring intense and time-consuming care, often increasing use of antipsychotic medication, and specific dementia-related professional expertise and communication skills [9], often leading to high physical and psychological workloads, strain, and burnout among professional caregiver [10–15].

Palliative rehabilitation as a non-pharmacological approach has in recent years become an important and integral part of care in nursing homes and long-term care facilities to prevent and manage BPSD and maintain an independent and meaningful life to the greatest possible extent, and consequently, improving quality of life and relieve the burden on care staff [16, 17]. Growing evidence suggests that horticultural activities and exposure to green spaces improves physical and mental health for all [18–20], and specifically for older adults [21–23]. Also, research indicates that there are additional benefits specific to PwD such as reduced agitation and use of psychotropic drugs, improved sleep and communication skills, and higher levels of social interaction and physical functioning [24–27], as well as ease the burden on staff and increase job satisfaction [28]. However, people suffering from severe dementia are mostly not able to plan and carry out activities, which will influence their ability to access and use gardens independently. Consequently, they rely on person-centered structured activity-programs and staff who are able to assist PwD in going outdoors, provide meaningful activities and appropriate level of assistance with activities [29]. A scoping review by Gonzalez et al. highlights the many different ways of facilitating nature-based interventions in dementia care, but also points out that there still is limited knowledge in this field of research [30]. Designing dementia-friendly gardens in nursing homes may support these nature-based interventions and many nursing homes already have gardens to be used by residents, visitors, and staff, of which some are designed specifically for PwD. However, evidence suggests that these gardens are underused, especially by those living with dementia [30–32]. Several reasons for this have been reported in the literature, such as inappropriate garden design, inaccessible outdoor spaces, organizational risk aversion, staff’s safety concerns, attitude, lack of training, skill, and knowledge, as well as institutional policy and lack of leader support [24, 30, 33]. Consequently, there is a lack of knowledge on how to implement gardens in nursing homes for PwD to ensure greater use by PwD and their caregivers, and consequently, greater palliative care.

### Aim

This paper will present the study protocol of the ‘Gardens for People with Dementia’ study. The aim of the study is to develop and implement a nature-based palliative rehabilitation intervention on how nursing home gardens can be used by PwD to improve a range of health outcomes. The paper includes a description of the study case, the co-design-based re-design of a nursing home garden and development of a nature-based palliative rehabilitation program, the implementation of the nursing home garden, and measurements used in the process- and effect evaluation of the study.

## Methods

### Cross-disciplinary research team

It is today expected that the healthcare sector uses evidence and cross-disciplinary knowledge in their decision-making, and that research from different fields is used to solve problems within healthcare [34]. This cross-disciplinary knowledge, however, is difficult to translate into specific solutions and is therefore rarely used in practice. In general, translating research into practice is challenging [35] and demands cross-disciplinary collaborations between researchers and practitioners to ensure knowledge to action. The difficulties in the implementation of gardens at nursing homes for PwD, requires researchers, landscape architects, occupational therapists and others interested in promoting garden use at nursing homes, to work interdisciplinary to ensure the translation of research into practice, as well as greater implementation and integration in the daily work and lives at the nursing homes. With this in mind, the present study is conducted by an interdisciplinary team of researchers and practitioners with a broad range of backgrounds. The team consists of a chief physician within a Dementia Clinic at a hospital, two landscape architects, one physiotherapist, one former nurse and now quality and development consultant within the health sector, and one public health researcher. All but the nurse are also researchers. This cross-disciplinary collaboration is expected to foster the translation of research into practice, and thus, greater implementation of the intervention.

### Study design

**Fig. 1:**
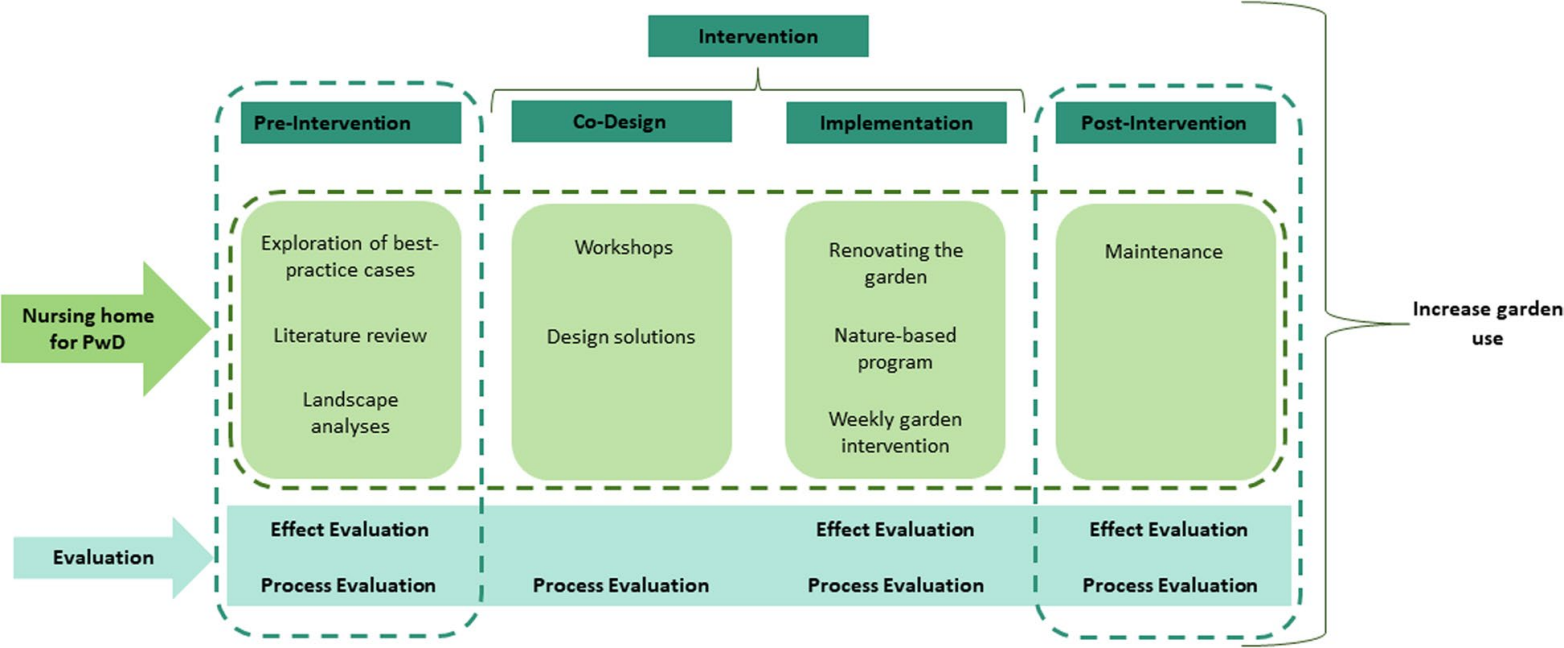
Illustration of study design

The study is a quasi-experimental single-cased case–control intervention study based at a nursing home for PwD. The study takes place between April 2022 and September 2023. The study is divided into four phases: 1) pre-intervention; 2) co-design; 3) implementation; 4) post-intervention (see Fig. 1). Information collected during the pre-intervention phase will be used to develop the co-design process (2.phase). Ideas and solutions from the co-design phase will be interpreted and implemented in the implementation phase (3.phase) in which the nursing home’s garden will be renovated and the nature-based palliative rehabilitation program implemented. The third phase also includes a case–control intervention where residents together with staff will participate in two weekly garden activities for one month. The final phase (post-intervention) will focus on the maintenance of the renovated garden as well as garden activities, to ensure that the project continues after the researchers have left the nursing home.

The evaluation of the study is based on a mixed methods design using quantitative and qualitative methods to conduct a comprehensive effect evaluation and a process evaluation. The effect evaluation will be conducted pre-intervention (baseline) and post-intervention (follow up), as well as right before (baseline) and right after (follow up) the nature-based palliative rehabilitation program to test the immediate effect of garden use. The process evaluation will be conducted during all four phases to describe how the intervention was developed and implemented.

### The case: nursing home for PwD

Recruitment of the nursing home was based on an invitation which was sent out to three nursing homes in Denmark. The nursing homes were invited as (1) only PwD resided there, (2) they were of acceptable size to enable effect analyses, and (3) their gardens seemed well designed to be used by PwD. The final nursing home included in this study was the only nursing home who answered the invitation with reasons related to issues with low garden-use, despite having a well-designed garden. The purpose was not to re-design a nursing home garden, but to gain knowledge on how low-cost design solutions can support a nature-based palliative rehabilitation program to improve heath and increase use of the garden at existing nursing homes challenged in using their gardens.

The intervention study takes place in a nursing home specifically for PwD, located in the southern part of Denmark. The nursing home has 52 residents with severe dementia living in small one-bedroom apartments including own bathroom and own kitchenette. Residents are allowed to keep pets in their apartment. The apartments are spread over three floors and assembled into smaller units with 8–9 residents in each unit, and a larger common area including a dining area, a kitchen, a living room, and smaller seating areas with windows overlooking either the garden or the parking lot. Half of the apartments are facing the garden and the other half are facing the parking lot. Meaning, that only half of the residents can see the garden from their apartment, whereas the other half can see the garden from the common area. Each unit has two balconies, one smaller facing the parking lot and one larger facing the garden. The balconies facing the garden each have an elevator which leads the residents to the garden at ground floor. The only access to the garden is through this elevator. Staff offices, meeting rooms, and larger kitchen are all placed at ground floor.

The nursing home has 75 care staff working on day-, evening- and night shifts. The staff consists mainly of health care workers and health care assistants, but also a few pedagogues and nurses. No occupational therapist or physiotherapist are working at the nursing home. One health care worker is working as an activity coordinator, planning different activities such as summer parties, walking trips, social dog visits. The nursing home also uses volunteers to help initiate activities with some of the residents. The staff has on average 18 sick leave days per year.

#### The garden

The nursing home is located within a social housing estate that was established between 1970 and 1980 and refurbished between 2006 and 2020 by C.F. Møller Architects, a well-acknowledged Danish architecture firm. C.F. Møller also designed the nursing home's garden and the adjacent green spaces, including a playground/activity area.

**Fig. 2:**
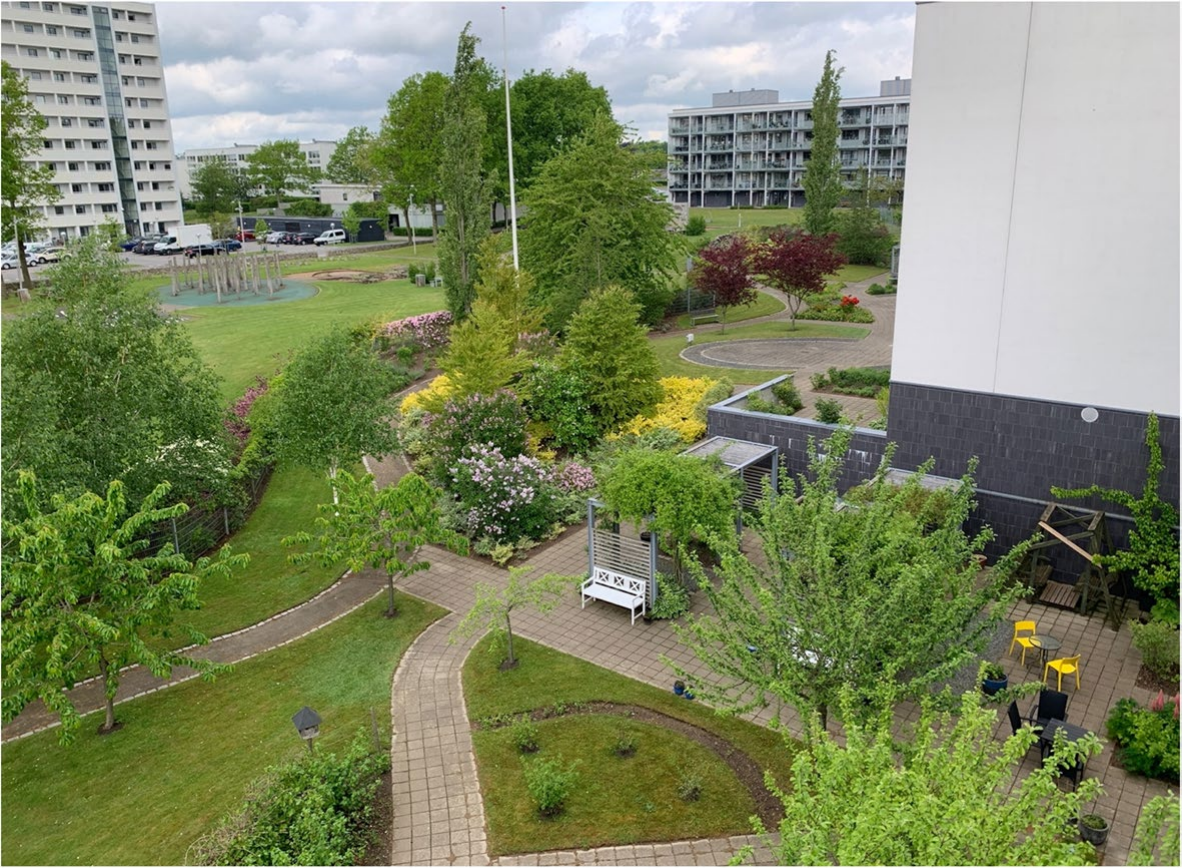
View over the garden from one of the balconies

The garden is oriented to the west, which provides sun from midday till sunset; however, this also means that the garden and the balconies facing the garden are exposed to wind most of the time, as this is the most prevalent wind direction in Denmark. The garden is in generally good condition and is very well maintained by volunteers from a local socioeconomic initiative (see Fig. 2).

**Fig. 3:**
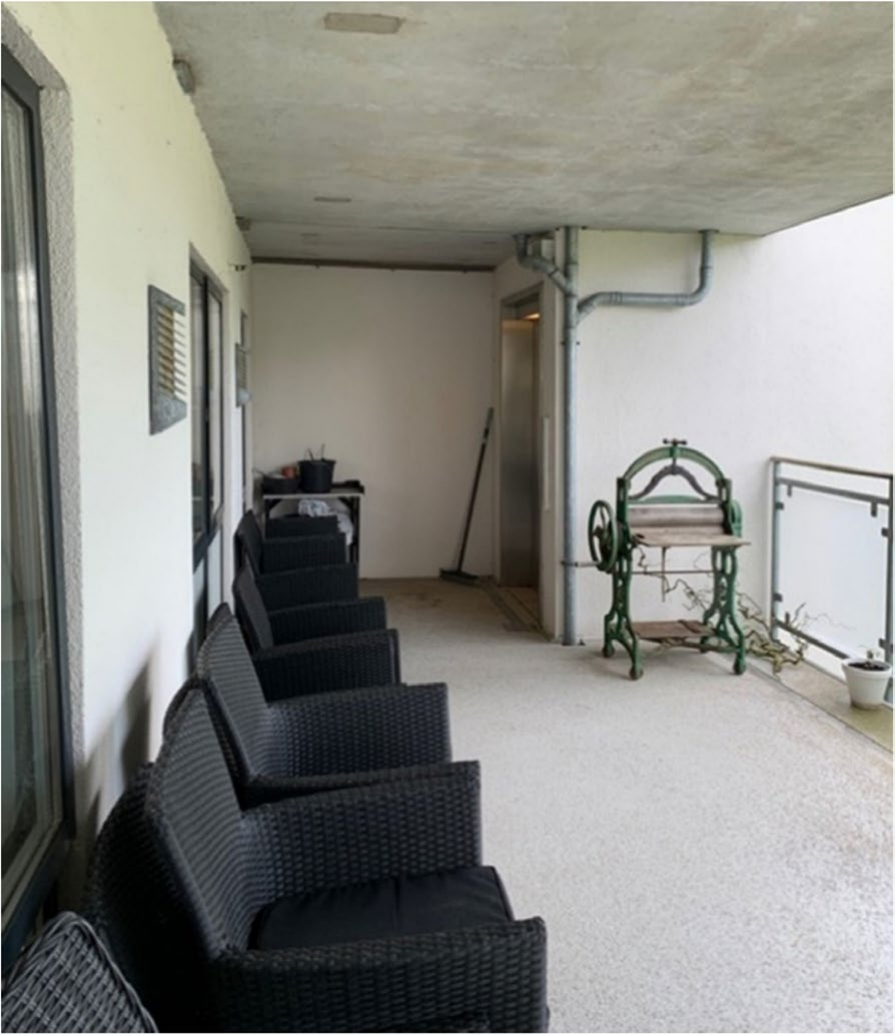
Balcony with garden access from the elevator to the right in the photo

As previously stated, elevators located on the balconies of each floor allow access to the garden. The balconies are made of concrete and are individually equipped on each floor, but the most of them have a few pots of seasonal flowers as well as an outdoor dining table and seating (see Fig. 3).

There are several seating options around the garden, although they differ in design and use, with some fixed and some moveable. The garden itself is approximately 3000 square meters in size and is separated from the surrounding green spaces by a metal fence that is sporadically covered with climbing plants. It consists of three smaller garden sections on different levels joined by a slightly sloping path in the west and steps in the east. Each level has its distinct character, which is described below.

##### Section 1 (south)

**Fig. 4:**
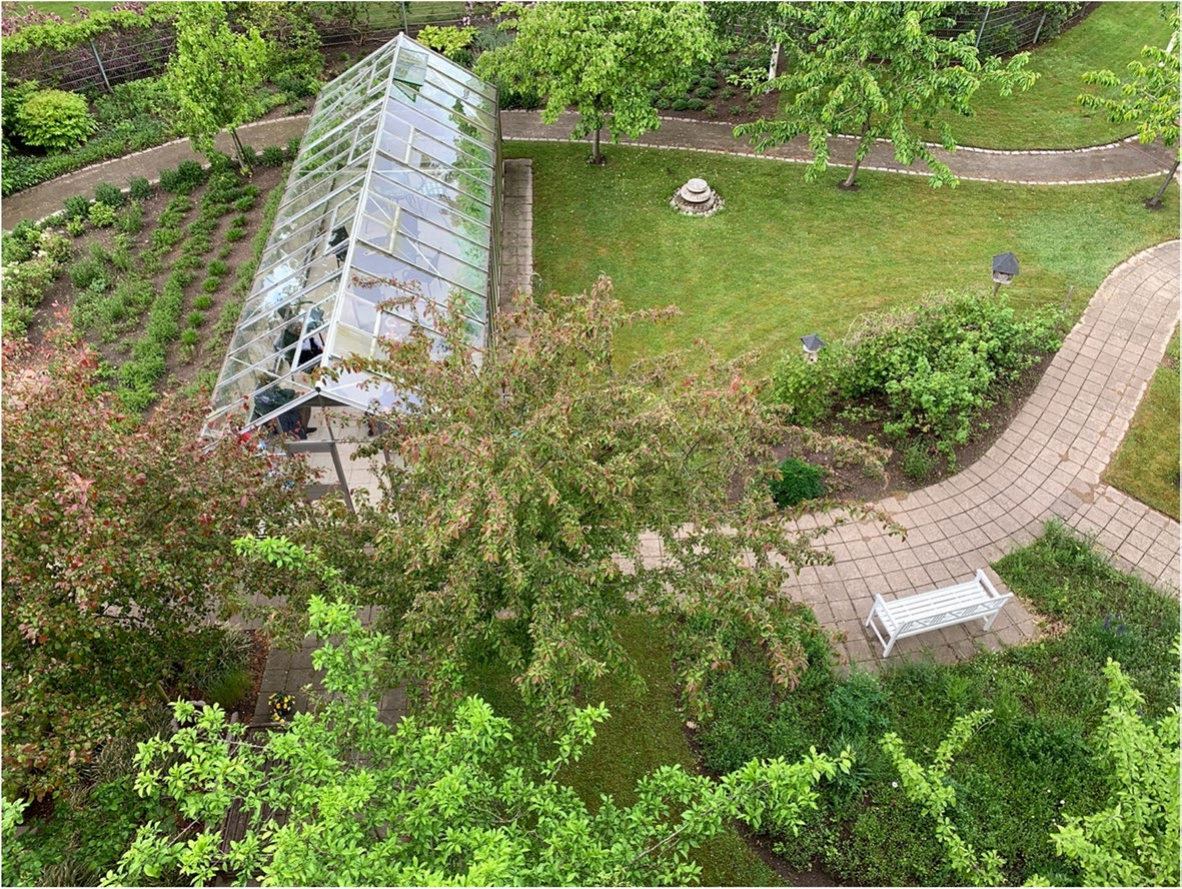
View from the balcony of 'Section 1' in the garden's southern section

This section of the garden is located to the south and includes open lawns, a large green house with tomato plants and grapes, fruit trees and other smaller trees, raised planting beds with herbs, a small terrace with seating, a large perennial bed, and pergola with a raised outdoor water basin (not in function) (see Fig. 4).

##### Section 2 (mid)

**Fig. 5:**
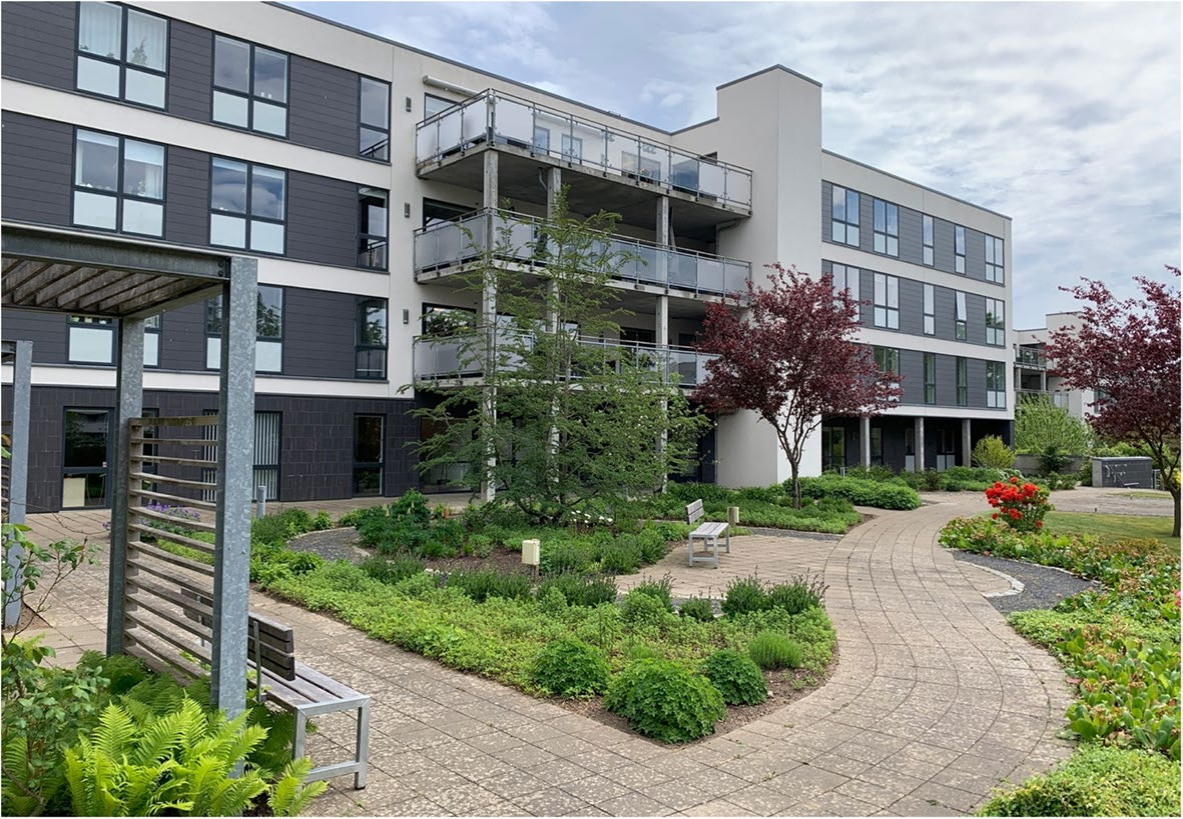
View from the pergola of the most of 'Section 2'

This area of the garden is made up of two bigger terraces surrounded by open lawns and beds filled with perennials and bushes, along with a few solitary trees of varying size. This section of the garden also contains a pergola as the one described above, as well as the same kind of raised outdoor water basin (yet again not in function) (see Fig. 5).

##### Section 3 (north)

**Fig. 6:**
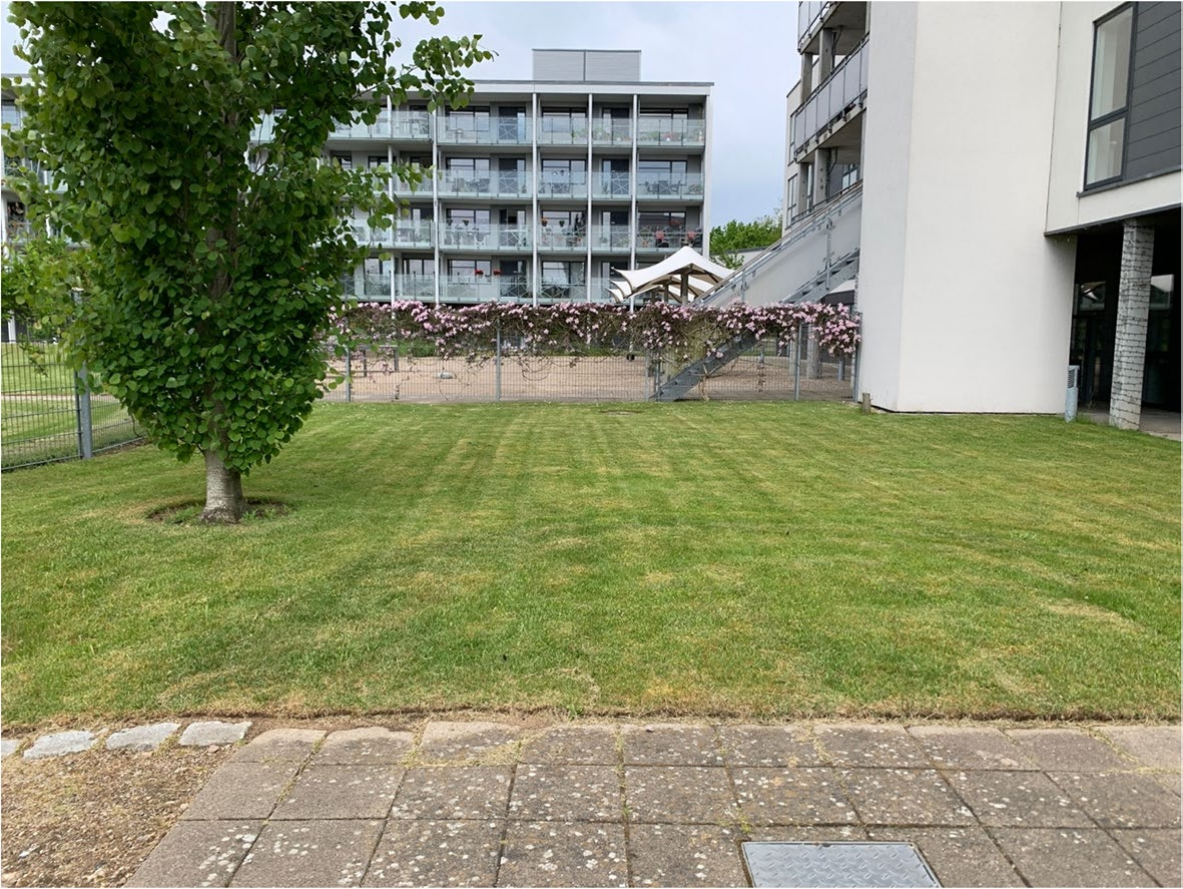
'Section 3' of the garden with a view of a playground behind the fence

This section of the garden is smaller than the two others and does only consist of an open lawn with a solitary tree and a rose bush (see Fig. 6).

### Pre-intervention

The pre-intervention phase is used as preparation for the two intervention phases – co-design and implementation. During this phase, several literature searches are conducted to gain up-to date knowledge on research and practical guidelines in three areas: 1) co-designing with people living with severe dementia in nursing homes; 2) effects of therapy gardens and horticultural therapy, on PwD; 3) Design of therapy gardens at nursing homes. Second, an exploration of best-practice cases is conducted to get insights into how other- and different types of nursing homes have succeeded in using their gardens in their everyday care of residents. The exploration has been conducted at two different nursing homes in Denmark, one being a small and private nursing home situated in the countryside next to fields and farms, and the other being a large public nursing home including a day care center situated in the city center of a larger town. At both nursing homes go-along interviews were conducted within their gardens, by audio recording the interviews while walking, and at the same time taking pictures of the garden and specific installations described during the interviews. Interviews were conducted with one leader and two staff at both nursing homes, and an additional interview was conducted with a volunteer at the public nursing home, since the private did not use volunteers. Interview questions included in the semi-structured interview guide are based on a framework presented by Grant et al. called ‘The Garden-Use Model’ which identifies five themes important for implementing or increasing garden use by residents with dementia living in long-term care facilities: 1) organizational policy; 2) staff attitude; 3) visual access; 4) physical access; 5) garden design [36] (see additional file 1). Third, a number of landscape analyses are performed to gain a better understanding of the garden's many spaces, functions, experiences, and usage options. These will, among other things, serve as the foundation for the garden's final design. The garden will be analyzed using the 'eye height analysis,' which focuses on what happens in the horizontal field of view in different situations (standing, sitting, etc.), as well as a customized version of the 'Kevin Lynch analysis,' which can help create an overview of the garden's various characteristics and how to navigate within it [37]. A 'visibility analysis' is also carried out to map different viewing zones in the garden (what can be viewed from which area). Furthermore, 'The eight perceived sensory dimensions' are utilized as an analysis tool to help map which room types are present in the garden and what characteristics they have [38], as well as an analysis of way-finding. Finally, a mapping of the garden's existing planting will be performed, which will serve as the foundation for the addition of new plants as well as provide an overview of the garden's diverse plantings in relation to future horticultural activities.

Knowledge from the exploration of best-practice cases, landscape analyses, and from the three literature searches are used to gain an understanding of the case, to inform the planning of the co-design and implementation processes, and to develop a *program.* A program is a list of functions that a certain site should have, based on the preferences, and needs of the users and/or client, as well as how the landscape architect/architect intends the site's users to interact with it. In this case, the project's landscape architects will use the Evidence-based Health Design in Landscape Architecture (EBHDL) process model [39, 40] to collect evidence on the target group (PwD), knowledge on the nature-human health relationship, the garden, and the use of nature, to develop a program with specific design criteria and design solutions for the garden.

### Intervention

The intervention is divided into two phases – a co-design phase and an implementation phase – will be described in detail in the following.

#### Co-design team

In the co-design phase, the research team will explore how the garden at the nursing home can be adapted, to support garden activities that can be experienced as meaningful to the residents (PwD). The strategy is based on involving the participants early, including staff, relatives, volunteers, and residents (PwD), to ensure a successful implementation and anchoring of the project [41] The co-design process is based on three tracks that will help to provide a participant's perspective:

1. Staff takes part in the project, experience being listened to, and understand the importance of their role in connection with the residents’ (PwD) use of the garden.
2. Participants are in an ongoing dialogue with each other about their wishes for the design of the garden and the opportunities for activities together with the residents (PwD).
3. The project is experienced as meaningful for those involved, based on a consideration that they through their own needs contribute with their perspective in relation to the connection between garden design and activities.

The participants in the project will be divided into three main groups, which in different parts of the intervention will be divided into subgroups or combined, where it makes sense. These groups are: (1) the co-design team, (2) PwD and their contact persons, (3) entire care staff in the nursing home.

The co-design team is composed of two managers and six care workers representing the six sections of the nursing home, as well as four relatives, two volunteers and a janitor. The organizations of the group should represent groups or people who reside in, work at, or otherwise contribute to life at the nursing home. Due to the high number of participants, cancellations from some participants will not be decisive for the outcome of the workshops. The managers at the nursing home will select five residents who are able to participate in the process and representative for the entire group of PwD at the nursing home. In addition to this, the research team strives to involve a larger group of PwD from the nursing home ad hoc, among other things through various garden activities. When PwD are involved in design research, it is important that they can attend activities together with a well-known contact person, who ensures their well-being [42]. Therefore, the project requires the PwD to participate together with a contact person (a relative or care staff). The staff counts the total group of care staff (*n* = 75) who are not already a part of the co-design group and consists of employees who manage care tasks for the residents (PwD) of the nursing home.

#### Workshops, meetings, and activities in the co-design phase

Results from the pre-intervention phase, as well as experiences within the research team, contributed to tools and recommendations for further planning, in the form of guidelines for the practical execution of workshops, meetings, knowledge sharing, and highly tailored activities for the PwD. The co-design phase is planned to include four workshops for the co-design group, one meeting for the staff, and a garden activity day for PwD. In addition, the co-design group and the rest of the care staff will receive homework on the development of garden activities between the workshops (see Table 1). Through workshops and staff meetings, the research team strives to promote an innovative and inclusive process that, through information flow from the staff group to the researchers, provides information that helps to concretize the design process and knowledge about the use of the garden.

**Table 1:** Overview of co-design activities

	**Workshop 1** Theme: The garden	**Workshop 2** Theme: Nature, activities, and human health	**Activities before workshop 3** Theme: Dialog between staff, co-design group, and PwD about experiences and meaningful activities	**Workshop 3** Theme: collection and qualification of ideas for activities	**Workshop 4** Theme: Presentation of design plan for garden
**The co-design group**	Talk: Gardens at care facilities Activity: The interaction between the garden and the activities and how the garden is perceived	Talk: Connection between human health and nature environments Activity: Starting the innovation process creating outdoor activities	Pictures, notes, and drawings from co-design group are collected to be used in workshop 3		Talk: Presenting drawings Activity: Commenting on drawings
**The staff**	Talk: Connection between human health and nature environments Activity: Nature-activity for stimulation of senses, physical activities, and leisure		Pictures, notes, and drawings from staff are collected to be used in workshop 3		Talk: Presenting drawings Activity: Commenting on drawings
**PwD**			Garden day with four-five activities for PwD Observations from their interaction with the garden and activities		

The aim of the workshops is to learn about the garden’s potential and to participate to a lengthy procedure of identifying and developing garden activities all year long. Between the workshops, the co-design group keeps working with the staff group to create ideas for garden activities and identify potential obstacles to utilizing the outside space in daily life. The staff meetings' two main goals are to ensure sustainability and anchoring after the project is over, and to include the staff in the co-design process. Two staff meetings with the same agenda are scheduled, to improve the chance that each member of the staff can make it to one of the sessions. To improve awareness of the garden's relevance and to contribute to a shared appreciation of the significance and potential of the outdoor area in staff and residents' daily lives, the staff is educated on the advantages of nature for physical, mental, and social health during the meetings. Additionally, the staff is invited to various sensory garden experiences that aim to offer a sense of well-being in the garden, as well as some garden activities that aim to foster learning by experience. As part of the co-design phase, the research team will host a garden day for the residents (PwD) and their contact person, with the aim of testing four-five garden activities and observing the behavior of PwD, with the purpose to gather information of the PwD’s experience of garden activities. These activities will partially be developed through the co-design phase. Activities are important in relation to the participation of PwD, particularly activities that induce enjoyment and well-being for PwD [43]. Daily tasks, hobbies, play, and other activities that have been a part of PwD’s life, frequently affirm their identity. Due to the PwD’s varied cognitive abilities, it is important for researchers to be adaptable, patient, and to provide different forms of participation [42]. Therefore, activities for the garden day will differ in terms of both the cognitive requirements for participation as well as the physical and social requirements.

#### Re-design of the garden

The landscape architects in the project will make a program with specific design criteria and design solutions based on landscape analyses, the outcomes of the exploration of best-practice cases, the literature searches, and the co-design process. This program will form the base of the actual re-design of the garden. Staff and the co-design group will be able to provide feedback on the final design during workshop 4, however, it is important to note, that all their wishes for the garden will not necessarily be fulfilled. The actual garden renovation will be carried out by external partners (for example, landscape gardeners) during winter season, based on the design provided by the landscape architects.

#### Implementation phase

During this phase, the new garden design is implemented. To ensure the staff's interest in garden activities in a broader sense and their willingness to accept responsibilities, a garden team is formed among them. The care staff is extremely important to involve and inform throughout the project period, as it is this group that, after completing the project, must ensure the continued use of the garden through meaningful activities.

Integrating garden activities into the nursing home's culture is one of the objectives of the implementation phase. Staff of three out of the six units at the nursing home will be required to facilitate garden activities for all residents at the three units two times a week for one month. Care staff will be asked to talk about practical experiences in the garden as they accumulate greater expertise throughout the implementation period. As a result, they become better at facilitating garden activities, and the new knowledge may become a part of their professional identities. The co-design group will simultaneously evaluate the tested garden activities, and a yearly cycle of events is planned for the PwD, their relatives, and staff members during all the seasons and special occasions. The project will require the creation of brief activity descriptions (idea cards), and examples of how staff and PwD might contribute to various activities must be described. The purchase of supplementary equipment (pruning shears, warm blankets, firewood, plant tables, pots, flower seeds, etc.) will enable the staff to begin garden activities with the PwD. The co-design team and staff will attend a follow-up workshop to discuss how gardening activities can be practically incorporated into daily care and fulfill the needs of PwD, especially those related to BPSD.

### Post-intervention

In the post-intervention, it is crucial to make sure that the nursing home staff anchors and develops relevant garden activities. Short lectures or workshops on using the garden and developing new activities are offered during monthly staff meetings, when the staff's experiences are included, and new ideas are produced. To ensure that activities in the garden are maintained all year long, this process will be initiated by the nursing home's garden team, who will also have the chance to communicate with the research team for a full year after the project is finished. To further the project's initiatives for generating experiences and long-term anchoring of activities in and usage of the garden, the garden group also passes significant experiences to the research group.

### Evaluation measurements

As described previously, the study consists of an effect evaluation across four data collection time points, and a continuous process evaluation across all four study phases. Each evaluation has their own data collection methods and measures, described in more detail below.

#### Effect evaluation

The aim of the effect evaluation is to examine the use of – and the effect of using the renovated and implemented garden for residents and care staff. The pre- and post-intervention effect evaluation will consist of: 1) systematic objective observations of garden users; and 2) questionnaire administered to staff on staff burnout and garden use. The pre- and post-implementation effect evaluation will consist of: 1) the The Neuropsychiatric Inventory Nursing Home version (NPI-NH) clinical instrument on residents’ BPSD symptoms and staff’s work disturbances; and 2) medication use.

##### Pre- and post-intervention

Systematic observations are conducted using a camera solution called HallMonitor developed by Webitall Aps [44]. The HallMonitor-system uses a cloud-based software platform and a number of cameras mounted on the wall. Each camera unit is configured to take a picture every half hour within a specified time-period, for example, from 6 am to 11 pm. HallMonitor uses a GDPR compliant design in which the camera takes a picture and immediately sends it through an encrypted 3G or 4G connection to a cloud-based platform storage which only Webitall has access to. The original picture is deleted from the camera unit after it has been sent to the platform. An automated script on the software platform processes the pictures automatically through a neutral network, which counts all the people in each picture whereafter the picture is being blurred. This process takes less than 10 min to complete. The unit has its own cellular data connection and only requires power to operate, making it easy to use in different settings. The camera system is so far mainly used by municipalities and sports associations to collect data on how different sports facilities are used by the public. This will be the first time that the system is used within nursing homes. Camera observations will be done for one month during August 2022 (pre-intervention), and at the same time one year later (August 2023) during post-intervention to systematically assess whether the intervention will increase the use of the garden. Cameras are placed to collect data from 6 am in the morning until midnight for 30 days, taking a momentary picture every 15 min. Four cameras are placed inside the nursing home, facing out into the garden, and one camera is placed inside the green house, to ensure that the whole garden is captured on camera. Additionally, one camera is placed on each balcony facing the garden, to detect any use of the balcony to get into the garden, giving a total of six cameras. The compiled data makes it possible to determine the number of users at different times and days of the week and month, as well as assess which parts of the garden are being used.

The questionnaire administered to staff related to staff burnout is based on Pines’ scale measuring a person’s feelings at work [45]. The scale consists of 21 items with responses stated on a seven-point scale going from ‘never’ (1) to ‘always’ (5). A person is regarded at risk of developing physical-, emotional- and/or mental exhaustion if they score between 3.0 and 4.0, whereas > 4.0 is considered experiencing and exhibiting symptoms of burnout [46]. Pines has tested the instrument for reliability and validity [45] and Åström et al. showed in their study high internal consistency and consistency between burnout scores and separate performed interview questions on burnout [47]. The instrument has previously been used in several studies [46–50], however, the instrument has, to our knowledge, not been previously used in a Danish context, and was therefore translated into Danish by the research team. The translation was performed by two researchers, who afterwards compared the translations and agreed on a common translation in case of inconsistencies. Additional questions were included in the questionnaire regarding staff’s garden use and attitude towards garden use (see Additional file 2). The questionnaire will be administered to staff through an e-mail send by the nursing home manager during July 2022 and again in July 2023. The staff will be given two weeks to complete the questionnaire and will be receiving a reminder e-mail after one week.

##### Pre- and post-implementation

Several information on residents agreeing to participate in the implementation phase will be collected during the pre-implementation phase. Information include sex, age, ability to walk independently, with assistance, or wheelchair bound, and the Mini Mental State Examination test (MMSE) [51] to assess the progression of the dementia disease. The MMSE is the most frequent and globally used instrument, takes 5–10 min to administer and is tested for its reliability and validity [52, 53].

The Neuropsychiatric Inventory Nursing Home version (NPI-NH) will in the study be used to evaluate the effect of the intervention on PwD’s BPSD symptoms. This instrument is currently the most used and recommended tool for people living in nursing homes [54], it has been previously validated and has been established in several languages [55]. NPI-NH is developed to assess neuropsychiatric symptoms (BPSD) in people with mild, moderate, and severe dementia by care providers through a retrospective and semi-structured interview. The instrument consists of 12 different symptoms common in PwD: hallucinations, delusions, depression/dysphoria, anxiety, agitation/aggression, elation/euphoria, disinhibition, irritability/lability, apathy/indifference, aberrant motor behavior, appetite/eating change, and nighttime disturbances. First, a screening question is asked to determine if the 12 behavioral changes are present or absent, if changes are present, probing questions are assessed related to frequency of the behavior, severity, and how much the behavior is disturbing for the care provider [55]. Additionally, information on participants’ medication use is registered. The questions will be asked by a trained researcher interviewing each care professional identified as the primary care provider for the participating PwD. The interview sessions will be conducted as part of the daily work in the nursing home. The sessions will be delivered pre-implementation in March 2023 and post-implementation in May 2023. Reason for the short timeframe between pre- and post-implementation is because it is expected that participants’ dementia is progressing rapidly since most participants have severe dementia. Whereas the pre- and post-intervention evaluation does not require such short timeframe since the data collection is designed as repeated cross sectional.

#### Process evaluation

The aim of the process evaluation is to evaluate how the intervention has been implemented [56]. In the pre-and post intervention phase, interviews will be conducted with selected co-design team members. The interviews are designed as two focus group interviews, one including care staff and the other including relatives and volunteers. No interviews are done with residents, based on recommendation from the nursing home, due to their severe dementia. The first focus group interview will focus on the different experienced challenges before the co-design process, and the expectations for the coming development of the garden. The second focus group interview will provide insights into the development and implementation of the garden, experienced acceptability, appreciation, and appropriation of the garden design and intervention. The focus groups will be conducted locally at the nursing home and will be audio-recorded. An interview guide will be used to guide the semi-structured interviews based on the ‘Normalization Process Theory’ of implementation (see additional file 3). This theory focuses on four factors relevant for successful implementation: Coherence, Cognitive participation, Collective action, and Reflexive monitoring [57]. Two semi-structured single interviews will additionally be conducted with the nursing home manager and activity coordinator pre- and post-intervention to assess organizational challenges, staff attitude and physical challenges. The interview guide for the single interviews are based on the same framework by Grant et al. presented in the pre-intervention phase, called ‘The Garden-Use Model’ [36] (see additional file 1). During the co-design phase, field notes will be collected by one research team member not participating in the co-design workshops, to gain an understanding of the context and the co-design process explored, as well as challenges in co-designing with PwD. The filed notes will be guided by the Normalization Process Theory already presented (see additional file 3).

### Data analysis

#### Effect evaluation analysis

##### Pre- and post-intervention

The systematic camera observations will provide information on how many people are using the garden and which parts of the garden they are using before and after the intervention, 24/7 and for a whole month. This will provide information on whether more people are using the garden after the intervention and if they are using the renovated parts of the garden. As the camera is taking four pictures per hour, the amount of data at the end of each data collection will be 20,160 blurred pictures. For each picture, the cloud-based software platform will provide the number of people in the picture, which will be a data point in the dataset. Consequently, the dataset will consist of 20,160 datapoints sorted by date at baseline and the same amount again at follow up. The blurred pictures are used to identify which areas of the garden are being used. The garden will be divided into several zones and each zone will be given a number. A student assistant will go through all pictures and classify in which zones people are observed and how many people are observed in the zones. The differences between pre- and post-intervention observation data will be analyzed as repeated cross-sectional measures as person identification is not possible.

The collected survey data on staff burnout and use of the garden completed by staff, will be uploaded into the statistical software IBM SPSS Statistics version 28. The differences between pre- and post-intervention will be analyzed as repeated measures comparing the degree of job satisfaction, use of garden, and attitude towards use of garden. This will provide information on whether the intervention has decreased staff burnout and has changed their use of the garden and their attitude towards using the garden in the daily care of PwD.

##### Pre- and post-implementation

The completed NPI-NH instrument will be converted into a scoring sheet which will provide a score on four different parameters for each of the 12 behavioral symptoms. These four parameters are: 1) frequency; 2) severity; 3) overall score for the domain (frequency x severity); 4) work disturbances for staff. An overall score is being calculated by adding up the 12 behavioral symptoms. The score on work disturbances is not included but will be calculated separately as an overall score on work disturbances, by adding up the scores for the 12 behavioral symptoms. The collected information on medication use is combined with the NPI-NH scores for each participant. The differences between pre- and post-implementation will be analyzed as repeated measures to assess any positive or negative change in BPSD-symptoms, work disturbances and medication use for the intervention group and control group and their respective primary care provider.

This is a real-world quasi-experimental study assessing the implementation of an activity to improve certain health outcomes. As this is a single-case exploratory study, sample size calculations are not applicable. The number of participants is all residents of the nursing home and their care staff. We do not expect generalizable results, but focus on the exploration of implementation.

#### Process evaluation analysis

The focus group interviews, and single-interviews will be analyzed as a whole using a mix of text-based content analysis and thematic analysis [58]. Relevant themes connected to how the process was experienced by the three focus group participants and leader will be extracted to identify barriers and facilitators during all stages of the co-design process as well as the implementation phase. Field notes collected during the co-design workshops will be analyzed using thematic analysis to extract relevant themes identifying barriers and facilitators to co-designing with people with severe dementia, their relatives, and staff at nursing homes.

## Discussion

The aim of this paper was to present the study protocol for the ‘Outdoor Spaces for People with Dementia’ study, including a description of the case, the intervention – divided into a co-design phase and implementation phase – and measurements to be used in the effect evaluation and process evaluation of the study. The study is a quasi-experimental single-cased case–control intervention study conducted at a nursing home for PwD, in which a co-design approach and implementation process will be used to develop and implement a highly tailored therapeutic garden. Designing this study was complex as there are many factors which might influence the results of such an intervention. This required a multitude of decisions that will yield a range of benefits as well as challenges discussed below.

### The interdisciplinary collaboration

Challenges in the implementation of nature-based palliative rehabilitation programs in nursing homes for PwD call for a different approach than previously used [24, 31–33]. Research institutions and authorities encourage and emphasize interdisciplinary collaborations and research of dementia care and rehabilitation [59]. The present project suggests a multi-disciplinary approach involving many different professionals and practitioners, which is a particular strength of the study. The study was planned and will be conducted as an equal partnership between researchers and practitioners from multiple fields, each using their respective competences to share ideas and complete the multitude of different tasks in the study. Even though other studies have shown that sharing design and planning experiences across different professions will increase quality and flexibility, improve efficacy, and stimulate appropriate use of resources, the same literature also suggests some difficulties in multi-disciplinary collaborations with balancing many different interests [60]. This has not been an issue in the present study yet, but difficult experiences from previous collaborations makes this an important focus area of the present project, which will be dealt with through continuous open discussion between all members of the research team throughout the entire project.

### Co-designing in long-term care facilities for PwD

The intervention process of this project consists of a co-design phase which will be used to develop a tailored intervention that will be implemented in the implementation phase. Designing in a partnership between nursing homes, PwD and their relatives has proven to be a difficult but rewarding and effective approach for addressing local needs in design projects [61]. However, most participatory research studies focus on people with mild or moderate dementia, and only few have experiences with people suffering from severe dementia [42]. Although previous research suggests that a participatory approach is important to the success of implementation [61], it also leads to a high degree of diversity in the intervention phase, making it difficult to fully plan the implementation of the garden and the evaluation of the project. Also, co-designing with people suffering from severe dementia is expected to be challenging. To be better prepared for unknown issues that will arise conducting participatory research with care staff, relatives and people suffering from severe dementia, we conducted a scoping review gathering guidelines, recommendations and research results on dos and don’ts in co-designing with people with severe dementia living in long-term care facilities. This scoping review will guide us in the planning and execution of the co-design workshops presented earlier (scoping review not published yet). Additionally, we conducted a pre-intervention study in which we explored two other long-term care facilities for PwD, who succeeded in implementing their garden in the daily care. This exploration will help us to gain insights into daily challenges and practical approaches used by the care staff and administration, which we can use to plan the co-design workshops and implementation of the garden. For example, we learned that the care staff’s attitude towards and skills for using the garden in the daily care of PwD is a crucial factor in the success of the implementation, which is why we have decided to have a greater focus on the care staff during the co-design workshops than first intended. This means that we will invite a greater number of staff to participate in the workshops and a smaller number of PwD. Lastly, the planned process evaluation will provide novel insights to the role and importance of the participatory co-design process for implementing needs and wishes of garden design and use at long-term care facilities.

### Learning from a single case

A single-case study such as this one is typically not appropriate for providing reliable results that can be generalized to a broader population. However, we decided on a single case due to the nature of the problem we are trying to investigate in this project. Providing recommendations for how to build gardens in long-term care facilities for PwD by applying previous research results on garden design is not new. However, the issue which we are trying to help to solve is, how to implement such a garden so that its use becomes an integrated part of the daily care of the residents. This is done by suggesting the use of a highly tailored nature-based palliative rehabilitation program. This focus requires an extensive evaluation allowing for detailed examination of all intervention stages, which is more difficult in large-scale research project. This requires a mixed methods approach combining an effect evaluation and a process evaluation using different types of data collection methods, which is a great strength of this study [62]. Flyvbjerg et al. argues that knowledge from single case studies is still valuable in the collective process of knowledge accumulation [63]. Consequently, the in-depth evaluation of this specific case study will lead to new knowledge that can be used to improve garden use at other long-term care facilities by using a tailored nature-based palliative rehabilitation program. In fact, one goal of the project team is to create a booklet, based on previous literature, results from the pre-intervention study, and experiences from this study, which provides tools and recommendations for other nursing homes on how to improve garden use.

## Supplementary Information

**Additional file 1. **(Single Interview guide): Interview guide based on The Garden Use Model.

**Additional file 2. **(Staff burnout questionnaire): Modified questionnaire on staff burnout, based on Pine’s scale and additional questions on garden use and attitudes.

**Additional file 3. **(Focus Group Interview- and Field Note Guide): the same guide is used for both focus group interviews and field notes, based on the Normalization Process Theory.

## Data Availability

Not applicable.

## Abbreviations

BPSD: Behavioral and Psychological Symptoms of Dementia
PwD: People with Dementia
NPI-NH: The Neuropsychiatric Inventory Nursing Home version
MMSE: Mini-Mental State Examination
