# Supplementary material for: Study design of an interdisciplinary and participatory nature-based palliative rehabilitation intervention in a Danish nursing home for people with severe dementia
Source: BMC Geriatr. 2022 Oct 23;22:819. doi: 10.1186/s12877-022-03513-6 (PMC9590121; doi:10.1186/s12877-022-03513-6)
Supplement: Supplementary file 1 — Additional file 1. (Single Interview guide): Interview guide based on The Garden Use Model. [file 12877_2022_3513_MOESM1_ESM.docx]

Single Interview Guide

Based on the ’The Garden-Use Model’ (Grant CF, Wineman JD. (2007) The garden-use model: An environmental tool for increasing the use of outdoor space by residents with dementia in long-term care facilities)

| **Theme** | **Research question** |
| --- | --- |
| **Organizational policy** | Defined as the stated goal of the facility regarding use of the outdoor space by residents with dementia.  This stated goal was founded on the following: a mission statement or similar document presented by the facility, available literature and brochures, the education and training of staff, use of volunteers, and programming philosophy. |
| **Staff Attitudes** | The overall staff mindset regarding the importance of the available outdoor space and the benefits that spending time outside offer residents. |
| **Visual Access** | Defined as encompassing views to the garden entry and  to the garden from the interior of the facility |
| **Physical Access** | Defined as the entry from the unit to the outdoor space including the  immediate area surrounding the threshold both inside and outside. |
| **Garden Design** | The spatial layout of the garden, circulation routes, and the features or amenities within the space, including pertinent distances between them, and the garden access. |
