# Supplementary material for: Study design of an interdisciplinary and participatory nature-based palliative rehabilitation intervention in a Danish nursing home for people with severe dementia
Source: BMC Geriatr. 2022 Oct 23;22:819. doi: 10.1186/s12877-022-03513-6 (PMC9590121; doi:10.1186/s12877-022-03513-6)
Supplement: Supplementary file 2 — Additional file 2. (Staff burnout questionnaire): Modified questionnaire on staff burnout, based on Pine’s scale and additional questions on garden use and attitudes. [file 12877_2022_3513_MOESM2_ESM.docx]

**Questionnaire on staff burnout and garden use**

(Åström et al. 1991, Staff burnout in dementia care-relations to empathy and attitudes)

| **Question** | **1 (Never)** | **2** | **3** | **4** | **5 (Always)** |
| --- | --- | --- | --- | --- | --- |
| To what extent do you experience stimulation in your work? |  |  |  |  |  |
| To what extent do you experience satisfaction in your work? |  |  |  |  |  |
| To what extent can you respond to expectations from the patient’s relatives? |  |  |  |  |  |
| To what extent do you feel satisfied in contact with demented patients? |  |  |  |  |  |
| To what extent do you experience the contact with a confused patient as stimulating? |  |  |  |  |  |
| To what extent do you experience the contact with a patient in his terminal state as stimulating? |  |  |  |  |  |
| To what extent does the work function well between day staff and night staff? |  |  |  |  |  |
| To what extent are your expectations from work satisfied? |  |  |  |  |  |
| To what extent do you experience satisfaction in your social life? |  |  |  |  |  |
| To what extent do relatives of demented patient’s respond to your expectations of them? |  |  |  |  |  |
| To what extent can you be helpful and see to the needs of a patient with dementia? |  |  |  |  |  |
| To what extent do you find your work rewarding? |  |  |  |  |  |
| When you feel satisfied in the contact with demented patients how satisfied do you feel? |  |  |  |  |  |
| To what extent can you respond to the expectations of your colleagues? |  |  |  |  |  |
| To what extent do you respond to the expectations of supervisory staff? |  |  |  |  |  |
| How often do you feel strain in the contact with demented patients? |  |  |  |  |  |
| Are you satisfied with your work goals? |  |  |  |  |  |
| To what extent do your colleagues respond to your expectations? |  |  |  |  |  |
| To what extent is your work-place ideal for the care of demented patients? |  |  |  |  |  |
| To what extent do you use the outdoor areas during working hours? |  |  |  |  |  |
| To what extent do you appreciate using the outdoor areas during working hours? |  |  |  |  |  |
| To what extent do you experience a positive change in residents’ behavior when being outdoors? |  |  |  |  |  |
| To what extent do you experience a negative change in residents’ behavior when being outdoors? |  |  |  |  |  |
| To what extent do you find it difficult to use the outdoor areas in the care of the residents? |  |  |  |  |  |
| To what extent are you satisfied with the outdoor areas in relation to the care of the residents? |  |  |  |  |  |
