# Supplementary material for: Study design of an interdisciplinary and participatory nature-based palliative rehabilitation intervention in a Danish nursing home for people with severe dementia
Source: BMC Geriatr. 2022 Oct 23;22:819. doi: 10.1186/s12877-022-03513-6 (PMC9590121; doi:10.1186/s12877-022-03513-6)
Supplement: Supplementary file 3 — Additional file 3. (Focus Group Interview- and Field Note Guide): the same guide is used for both focus group interviews and field notes, based on the Normalization Process Theory. [file 12877_2022_3513_MOESM3_ESM.docx]

Focus Group Interview- and Field Note Guide

Based on the Normalization Process Theory (Murray et al (2010) Normalisation process theory: a framework for developing, evaluating and implementing complex interventions)

| **Theme** | **Research questions** |
| --- | --- |
| **Coherence** (individually and collectively) | Relates to how the work that defines and organizes a practice/ intervention is understood, rendered meaningful and invested in, in respect of the knowledge, skills, behaviors, actors and actions required to implement it. |
| **Cognitive participation** | Relates to commitment to and engagement of participants with the intervention.  Do participants view the intervention as something worthwhile and appropriate to commit their individual time and effort (signing up) to bring about the intended outcome? |
| **Reflexive monitoring** | Relates to participants’ individual and collective on-going formal and informal appraisal of the intervention and its benefits for participants, in relation to realizing individual and organizational goals. |
| **Collective action** | Relates to the work that will be required of participants to implement the intervention, including preparation and/or training.  How far will existing work practices and the division of labour have to be changed or adapted to implement the intervention?  Is the intervention consistent with the existing norms and goals of the group, the workplace and overall organization (this is policy, practice and service user linked)? |
